# Multimodal Treatment Eliminates Cancer Stem Cells and Leads to Long-Term Survival in Primary Human Pancreatic Cancer Tissue Xenografts

**DOI:** 10.1371/journal.pone.0066371

**Published:** 2013-06-18

**Authors:** Patrick C. Hermann, Sara M. Trabulo, Bruno Sainz, Anamaria Balic, Elena Garcia, Stephan A. Hahn, Mallaredy Vandana, Sanjeeb K. Sahoo, Patrizia Tunici, Annette Bakker, Manuel Hidalgo, Christopher Heeschen

**Affiliations:** 1 Stem Cells and Cancer Group, Clinical Research Programme, Spanish National Cancer Research Centre (CNIO), Madrid, Spain; 2 Gastrointestinal Cancer Clinical Research Unit, Clinical Research Programme, Spanish National Cancer Research Centre (CNIO), Madrid, Spain; 3 Department of Molecular GI-Oncology, Ruhr-University Bochum, Bochum, Germany; 4 Nanomedicine Laboratory, Institute of Life Sciences, Bhubaneswar, India; 5 Department of Oncology, Siena Biotech S.p.A., Siena, Italy; 6 Children’s Tumor Foundation, New York, New York, United States of America; Technische Universität München, Germany

## Abstract

**Purpose:**

In spite of intense research efforts, pancreatic ductal adenocarcinoma remains one of the most deadly malignancies in the world. We and others have previously identified a subpopulation of pancreatic cancer stem cells within the tumor as a critical therapeutic target and additionally shown that the tumor stroma represents not only a restrictive barrier for successful drug delivery, but also serves as a paracrine niche for cancer stem cells. Therefore, we embarked on a large-scale investigation on the effects of combining chemotherapy, hedgehog pathway inhibition, and mTOR inhibition in a preclinical mouse model of pancreatic cancer.

**Experimental Design:**

Prospective and randomized testing in a set of almost 200 subcutaneous and orthotopic implanted whole-tissue primary human tumor xenografts.

**Results:**

The combined targeting of highly chemoresistant cancer stem cells as well as their more differentiated progenies, together with abrogation of the tumor microenvironment by targeting the stroma and enhancing tissue penetration of the chemotherapeutic agent translated into significantly prolonged survival in preclinical models of human pancreatic cancer. Most pronounced therapeutic effects were observed in gemcitabine-resistant patient-derived tumors. Intriguingly, the proposed triple therapy approach could be further enhanced by using a PEGylated formulation of gemcitabine, which significantly increased its bioavailability and tissue penetration, resulting in a further improved overall outcome.

**Conclusions:**

This multimodal therapeutic strategy should be further explored in the clinical setting as its success may eventually improve the poor prognosis of patients with pancreatic ductal adenocarcinoma.

## Introduction

Pancreatic ductal adenocarcinoma (hereafter referred to as “pancreatic cancer” or PDAC) is the fourth most frequent cause of cancer-related death world-wide [1], [2], [3] and is characterized by a high rate of metastasis and pronounced resistance to chemotherapy and radiation. Despite extensive research efforts over the past decades, little substantial progress has been made towards improving clinical endpoints [4]. Although the introduction of the anti-metabolite gemcitabine in 2007 has improved clinical response by reducing pain and weight loss [5], disease prognosis has remained extremely poor with a 5 year survival rate of ∼3–4% and a median survival period of 4–6 months [1], [6]. Indeed, several studies have consistently shown that gemcitabine treatment mostly targets differentiated cancer cells resulting in a relative enrichment of cancer stem cells [7], [8], [9]. For patients with metastatic disease, but good performance status, the recent combination therapy FOLFIRINOX (oxaliplatin, irinotecan, fluorouracil, and leucovorin) showed a significant survival advantage but with increased toxic side-effects [10]. Alternatively, the regimen of nab-paclitaxel plus gemcitabine showed substantial anti-tumor activity with more tolerable adverse effects in a phase I/II trial, warranting phase III evaluation [11]. However, in all these trials the majority of the patients ultimately succumbed from disease progression. Thus, the development of new anti-cancer therapeutics and/or new treatment modalities remains a high healthcare priority.

With increasing evidence supporting the existence of cancer stem cells, a new horizon is emerging in the development of therapeutic strategies against pancreatic cancer. Cancer stem cells represent a subpopulation of cells distinguishable from the bulk of the tumor based on their exclusive ability to drive tumorigenesis and metastasis. These cells also play a crucial and driving role in disease relapse [12], [13], [14], [15], [16]; therefore, the elucidation of the mechanisms underlying pancreatic tumorigenesis and especially pancreatic cancer stem cells is of crucial relevance for the development of more efficient clinically-available therapies. Indeed, we have recently developed novel approaches that both target cancer stem cells and overcome their mechanisms of chemo-resistance [9], [17], [18]. For example, we have shown that the self-renewal capacity of pancreatic cancer stem cells is dependent on both *Hedgehog* and *mTOR* signaling, and simultaneous targeting of these two pathways, in combination with Gemcitabine, represents a novel treatment strategy for epithelial cancers such as pancreatic cancer [9]. Building on these studies, we here investigate the applicability, safety, and potential for further optimization of this combination therapy approach in a large set of primary patient-derived tumors.

## Results

### Triple Therapy Markedly Reduces Tumor Size and Increases Survival

**Figure 1:**
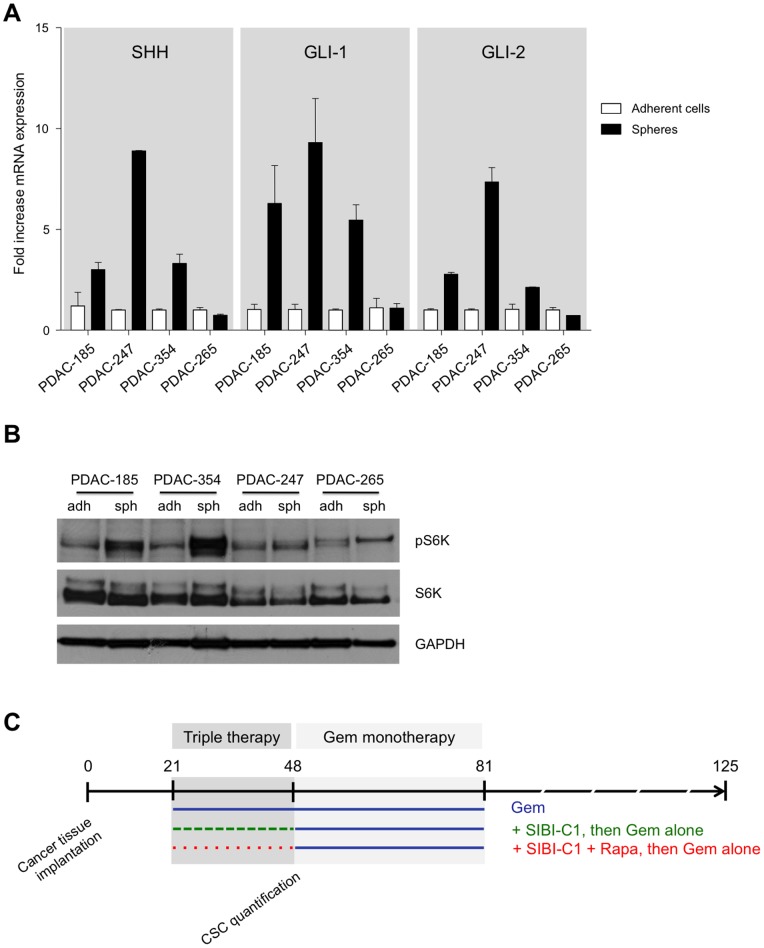
Targeting of sonic hedgehog and mTOR in pancreatic ductal adenocarcinoma. (**A**) Fold increase mRNA expression levels of SHH, GLI-1, and GLI-2 of sphere-derived vs. adherent cells. (**B**) Western blot analysis of mTOR pathway activity via the assessment of S6 kinase expression (upper panel) and phosphorylation (lower panel) in adherent primary cells versus stem cell-enriched sphere-derived cells. (**C**) Illustration of experimental setup. Duration of triple therapy is marked by a dark grey box (day 21 to 48), Gem monotherapy with a light grey box (day 48 to 81).

We have shown previously that sphere cultures of pancreatic cancer cells enrich for cancer stem cells [8], [9], [17], and that combined targeting of the Sonic Hedgehog (SHH) and mTOR pathways may offer a new therapeutic option. Here we verify in four distinct primary pancreatic cancer cell lines derived from patient tumors that cancer stem cell-enriched sphere cultures indeed show marked overexpression of SHH and the Hedgehog target genes GLI-1 and GLI-2 (**Fig. 1A**), as well as increased mTOR pathway activity (**Fig. 1B**). The subsequent *in vivo* evaluation of the combination therapy was performed in clinically most relevant models of patient-derived pancreatic cancer whole-tissue xenografts (see **Fig. 1C** for study design). Pieces of briefly *in vivo* expanded primary human pancreatic tumors containing heterogeneous populations of cancer cells including cancer stem cells [9] as well as stromal cells [7], pancreatic stellate cells, inflammatory cells, and extracellular matrix were implanted subcutaneously and orthotopically into immunocompromised mice. Tumor take rate was confirmed by tumor growth during two successive size measurements, and tumor-bearing mice were randomized for treatment. Subsequently, the tumors were measured once weekly either by caliper (subcutaneous tumors) or with a small-animal ultrasound imaging system (orthotopic tumors). As Gemcitabine (Gem) represents the current standard treatment for pancreatic cancer, we used Gem-treated mice as the reference group.

**Figure 2:**
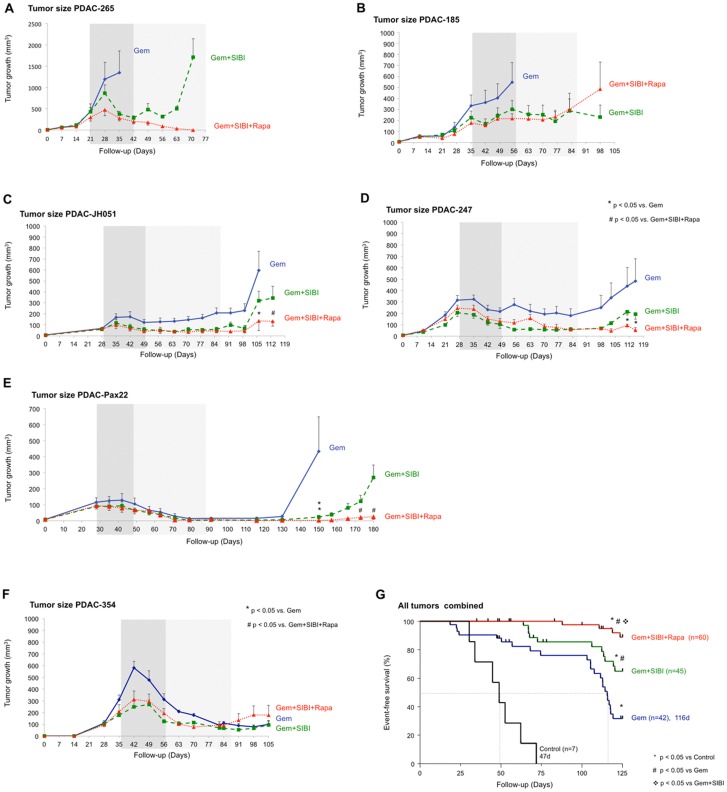
Combination therapy in a representative set of pancreatic ductal adenocarcinoma. (**A–F**) Tumor growth curves for primary whole-tissue xenografts PDAC-265, PDAC-185, JH051, 247, Pax22, and 354 implanted subcutaneously and orthotopically. Continuous line depicts Gem+vehicle, dashed line depicts Gem+SIBI, dotted line depicts Gem+SIBI+Rapa (n≥6 per group). (**G**) Kaplan-Meier Curve depicting cumulative survival time of all mice pooled by treatment group.

A set of representative tumors was selected based on their diverse response to Gem treatment [7]. PDAC-265 and 185 were highly resistant to Gem treatment, showing rapid tumor growth so that the first mice had to be removed from the study within 3 weeks of the start of the treatment (**Fig. 2A–E**) due to excessive tumor growth. In contrast, in tumors PDAC-JH051, 247, and Pax22, Gem treatment resulted in initial treatment response and disease stabilization; however, after the removal of chemotherapy, the tumors reproducibly started to re-grow (**Fig. 2C–E**). Of all the tumors investigated, only PDAC-354, which does not carry *Kras* mutations [19], showed significant response to Gem treatment until the end of the observation period (**Fig. 2F**) and closely mimicked the treatment response observed in the actual patient (data not shown).

**Figure 3:**
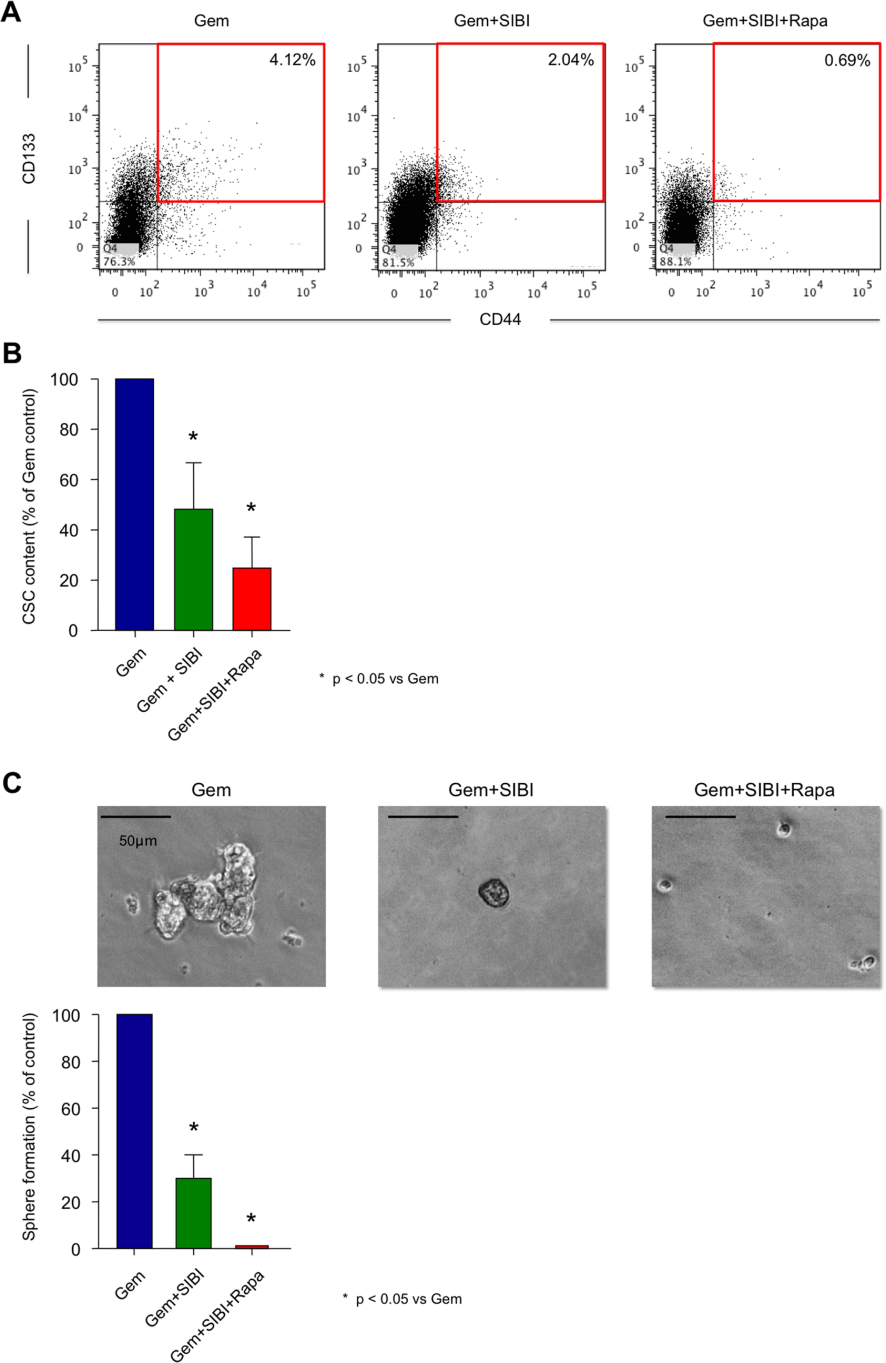
Effect of combination therapy on cancer stem cell content. (**A**) Representative flow cytometry plots and (**B**) quantification of cancer stem cell (EpCAM^+^CD133^+^CD44^+^) content of tumors in the respective treatment group (cumulative results of cells obtained from different xenografts). (**C**) Representative images and quantification of secondary sphere formation of treated PDAC-Pax22 tumors explanted at the end of the experiment (d200).

Importantly, we were able to improve treatment response by combining chemotherapy with the novel hedgehog pathway inhibitor SIBI-C1 (SIBI; Siena Biotech) [20]. SIBI strongly inhibits gene expression of SHH and downstream target genes such as GLI2 in primary pancreatic cancer cells *in vitro* (**Fig. S1A in File S1**). SIBI was administered for only 3 weeks to reduce potentially deleterious effects. Gem was given for a total time period of 60 days in accordance with common clinical practice (**Fig. 1C**). Due to the strong response to chemotherapy alone, co-treatment of mice bearing PDAC-354 xenografts with either SIBI did not show an additional effect at the level of tumor size (**Fig. 2F**) or survival (data not shown). For all of the other tumors, however, double treatment with Gem+SIBI led to a marked reduction in tumor size (dashed line, **Fig. 1** **&** **2**), significant delay in tumor growth, and thus significantly prolonged survival compared to mice receiving either no treatment or Gem+Vehicle (**Fig. 2G**). Importantly, however, tumors eventually relapsed limiting survival in mice receiving this double therapy. These data are in line with improved delivery of gemcitabine following depletion of protective stromal tissue [21]. As previously shown, inhibition of hedgehog signaling alone does not completely abrogate the cancer stem cell population (**Fig. 3A & B**) [**9**].

Since we have shown in comprehensive *in vitro* studies that cancer stem cells can indeed be eliminated by the addition of an inhibitor of the mTOR pathways [9], we next investigated the effect of a treatment regimen comprising Gem, SIBI, and established mTOR inhibitor rapamycin (Rapa) on our representative panel of primary pancreatic cancer tissue xenografts. Interestingly, we observed a very strong response to this triple therapy, resulting in disease stabilization or even regression in almost all tumors investigated (dotted line, **Fig. 1** **&** **2**). This translated into a significantly improved cumulative survival as compared to all other treatment groups (**Fig. 2E**).

### Combination Therapy Depletes Cancer Stem Cell Content and Alters Tumor Composition

In order to evaluate the *in vivo* effects of combination therapy on cancer stem cell populations, we explanted and digested representative tumors of each group after completion of the 3 weeks of triple therapy, and analyzed by flow cytometry the expression of surface markers previously linked to a cancer stem cell phenotype [8], [9], [13]. The percentage of EpCAM^+^CD133^+^CD44^+^ cells in Gem-treated tumors was regularly 2-3-fold higher as compared to untreated tumors [data not shown and [8]]. In contrast, Gem+SIBI already showed a slight decrease in cancer stem cell numbers as compared to Gem alone (**Fig. 3A & B**). Importantly, only the addition of Rapa to the treatment regimen virtually eliminated cancer stem cells from the tumor. Furthermore, upon termination of the study period (day 200) we investigated secondary sphere formation as a functional assay for cancer stem cell activity in PDAC-Pax22 tumors and observed that sphere formation capacity was slightly diminished for cultures derived from tumors treated with Gem+SIBI as compared to Gem alone. Interestingly, however, it was only after triple treatment that we could observe complete abrogation of sphere formation activity (**Fig. 3C**), suggesting that triple combination therapy had effectively depleted the cancer stem cell pool in the tumor.

**Figure 4:**
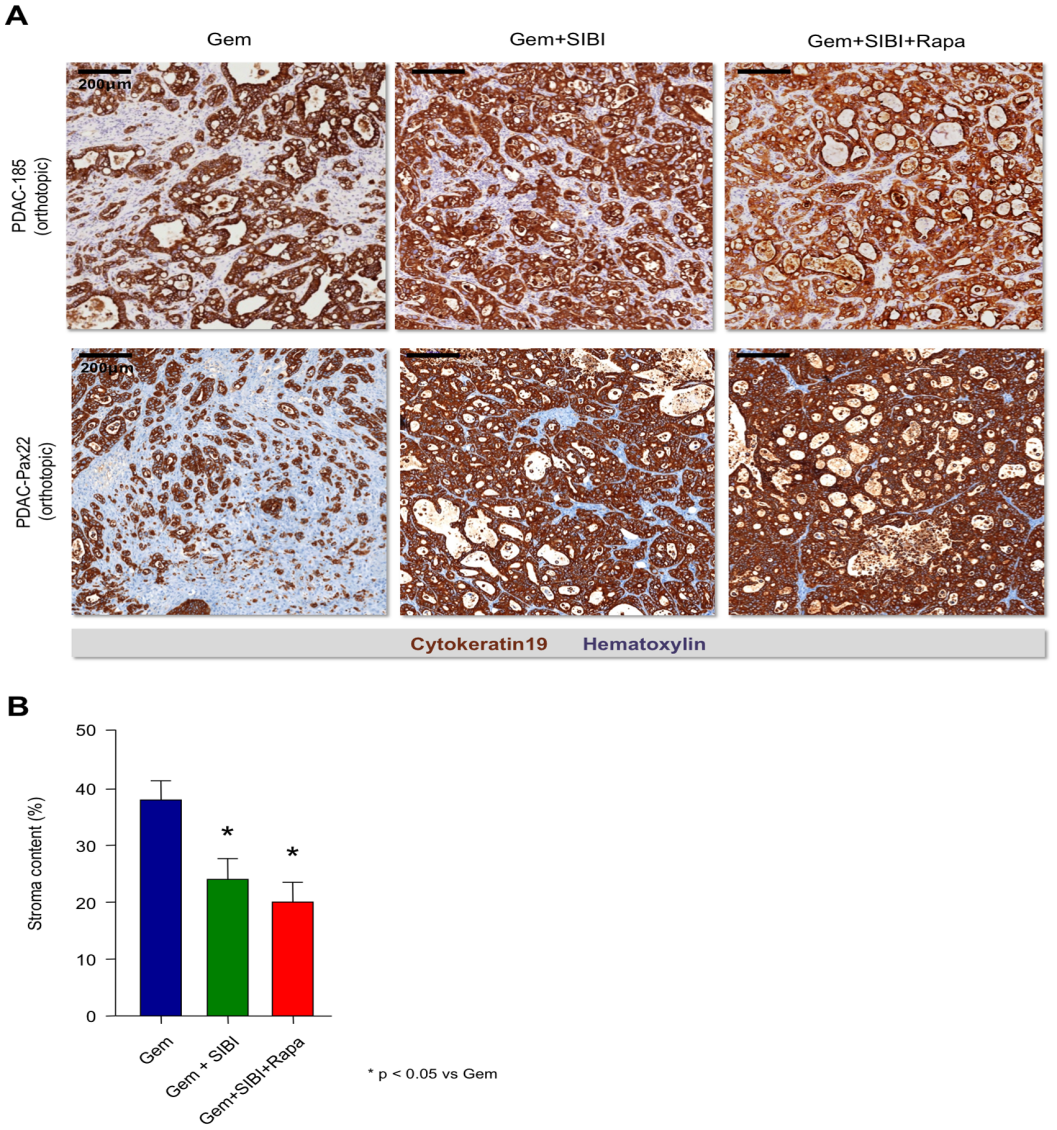
Effect of combination therapy on tumor composition. (**A**) Representative histological pictures showing stroma content in the respective treatment groups in gemcitabine resistant orthotopic tumors (**PDAC-185, upper panel**), (**Pax22, lower panel**). (**B**) Quantification of stroma content throughout the different treated xenografts.

More detailed histological investigation of the tumors showed that the different treatment regimens also modified the cellular composition of the tumor. While the primary tumor-derived xenografts used for this study displayed a reasonably high amount of stroma (35–70%) in the groups treated with Gem only (**Fig. 4A, left panel** **s**), the addition of a hedgehog pathway inhibitor markedly decreased the stroma content (**Fig. 4A** **, middle panels**), an observation that is well in line with previous published reports [21]. This effect was slightly more pronounced after the addition of Rapa (**Fig. 4A, right panel** **s**), and was statistically significant as compared to tumors treated with Gem alone (**Fig. 4B**). As expected, we observed the same effects after treatment of Gem-sensitive tumors (**Fig. S1B in File S1**). Interestingly, we observed similar effects in orthotopic tumors (**Fig. 4A**) as in subcutaneous tumors (**Fig. S1B in File S1**).

### PEGylation of Gemcitabine further Enhances the Effects of Combination Therapy

**Figure 5:**
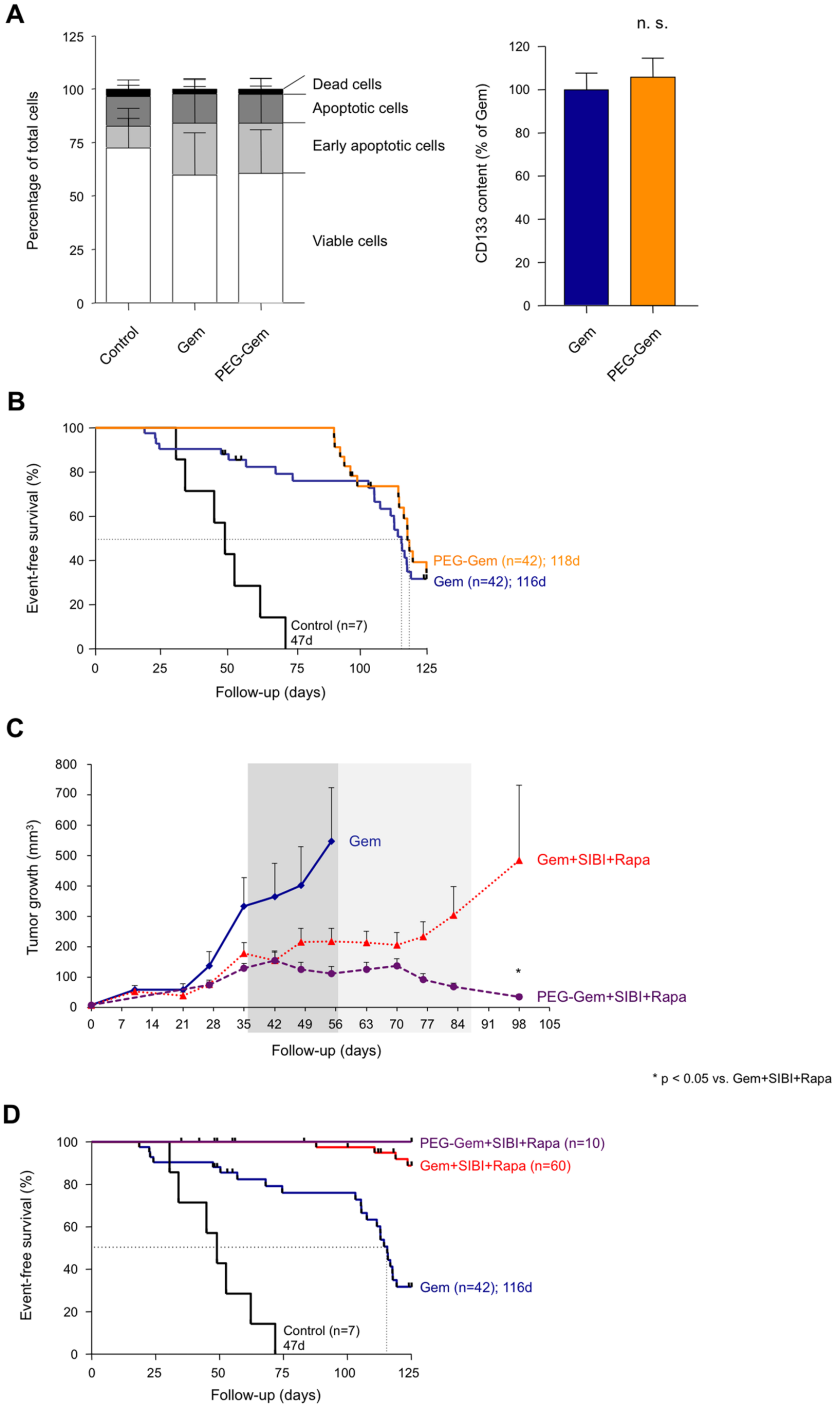
Comparison of the *in vitro* and *in vivo* effects of Pegylated Gemcitabine. (**A**) *In vitro* effects of Gem and PEG-Gem on apoptosis and cell death as well as CD133 expression (cumulative results of cells obtained from different xenografts). (**B**) Kaplan-Meier Curve depicting cumulative survival time of all mice pooled by treatment group. For illustrative purposes, selected survival curves of Fig. 2D are depicted again. (**C**) Tumor growth curves for primary whole-tissue xenografts implanted subcutaneously and orthotopically, respectively. Continuous line depicts Gem+vehicle, dashed line depicts Gem+ SIBI, dotted line depicts Gem+SIBI+Rapa. (**D**) Kaplan-Meier Curve depicting cumulative survival time of all mice pooled by treatment group. For illustrative purposes, selected survival curves of Fig. 2D are depicted again.

Since previous reports have shown that modifying the chemical structure of Gem by PEGylation leads to significantly increased circulation time and tissue penetration *in vivo* and may therefore be a novel option for the improved treatment of patients with (pancreatic) cancer [22], [23], we decided as a next step to determine the effects of PolyEthyleneGlycol-bound Gem (PEG-Gem) as the extended *in vivo* circulation time and higher tissue penetration of PEG-Gem may generate superior effects as compared to standard Gem. First, we evaluated the *in vitro* effects of PEG-Gem as compared to Gem on freshly isolated primary human pancreatic cancer cells. For this purpose, four matching primary cell cultures generated from *in vivo*-expanded pancreatic cancer tissues were treated for 48 hours with either standard Gem or PEG-Gem and were subsequently analyzed by flow cytometry for the induction of apoptosis or cell death, as well as for their cancer stem cell content. Regarding the percentage of apoptotic and dead cells, no differences could be observed between treatment groups (**Fig. 5A left panel** **, and data not shown**). In addition, we did not observe differences between standard Gem and PEG-Gem treatment regarding the content of CD133^+^ cells *in vitro* (**Fig. 5A right panel**).

Next we treated mice bearing orthotopic or subcutaneous primary tumor-derived whole-tissue xenografts with PEG-Gem, analogous to the treatment regimen for standard Gem. We selected tumors that showed insufficient response with Gem. While we did not observe a significant difference for median survival between PEG-Gem and standard Gem for these tumors (**Fig. 5B**), it is important to note that the onset of tumor-related death in mice treated with PEG-Gem was far later than with standard Gem (Time until progression: PEG-Gem 91d vs. Gem 19d). Encouraged by these promising results, we next replaced standard Gem treatment with PEG-Gem in the triple therapy approach (PEG-Gem+SIBI+Rapa) and evaluated the effects on tumor growth and survival in mice bearing PDAC-185 patient-derived xenografts. While in our initial *in vivo* studies Gem+SIBI+Rapa treatment led to significantly reduced tumor growth and short-term sustained disease as compared to standard Gem treatment (**Fig. 2D**), many tumors eventually relapsed. In response to treatment with PEG-Gem+SIBI+Rapa, however, we observed virtually complete regression of the tumors (**Fig. 5C**), which resulted in 100% survival until the end of the observation period (day 125) (**Fig. 5D**).

### Combination Therapy Shows no Significant Toxicity

**Figure 6:**
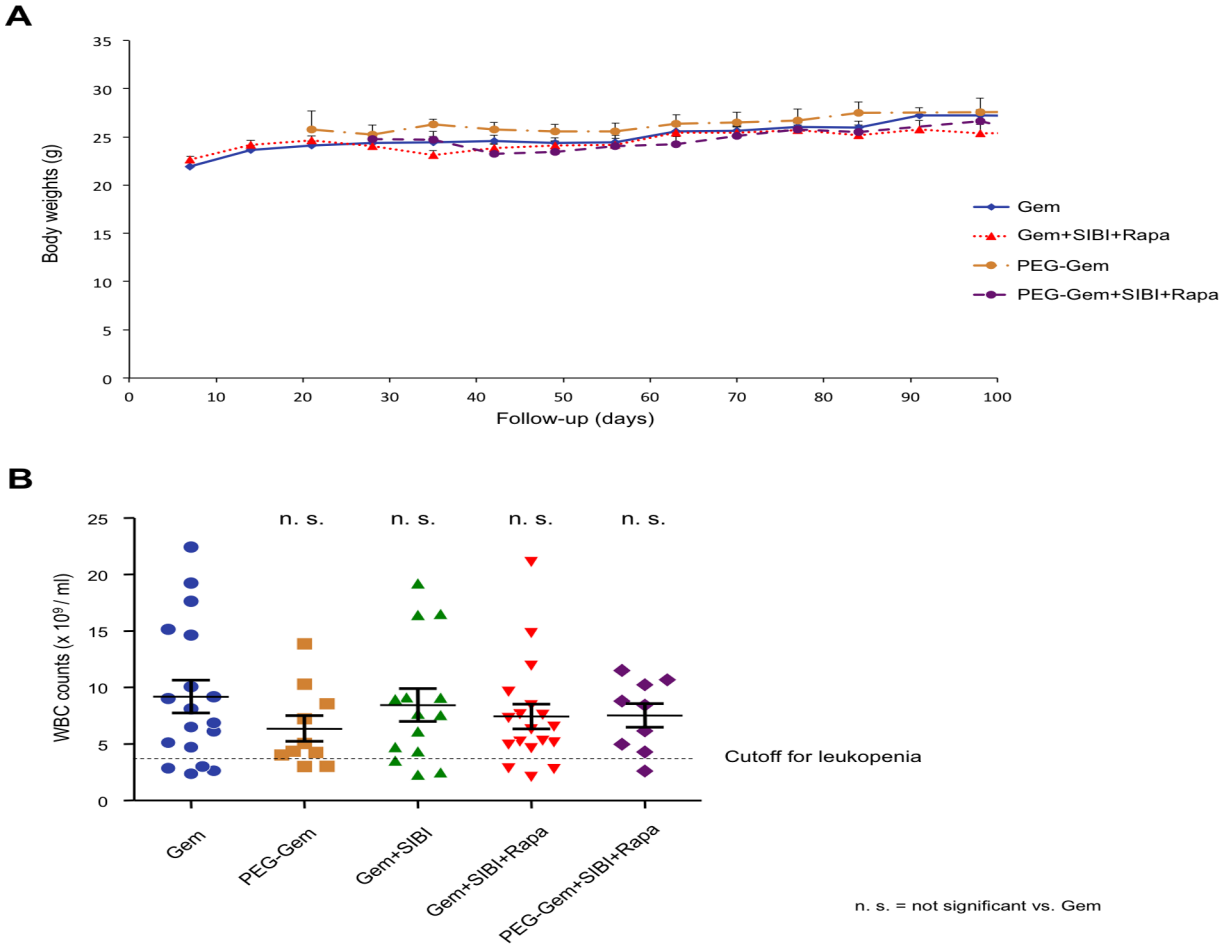
Assessment of in vivo biocompatibility/safety. (**A**) Body weights were recorded for all mice throughout the first 100 days of the experiment. (**B**) White blood cell counts of all mice were assessed at the end of the administration period of the triple combination.

Potential toxicity remains a major concern for combination therapy approaches. To assess cumulative toxicity of the administered treatments and their respective combinations, we recorded body weight for all treated mice on a weekly basis, starting on the day of randomization until day 100. Excluding cachexia as a potential treatment-induced side effect, no significant differences in body weights were observed between the treatment groups (**Fig. 6A**), Furthermore, in order to exclude potentially deleterious effects on the function of normal stem cells (e.g. in the hematopoietic system), we additionally monitored white blood cell numbers in the treated mice at the completion of the 3 weeks of single versus combined therapies. While at this point of the study the expected cumulative toxicity would be the highest, no significant reduction in white blood cell counts was observed in any of the treatment groups as compared to standard Gem treatment (p = 0.792) (**Fig. 6B**), suggesting no extensive alterations of hematopoietic stem cells by the triple combination treatment. Interestingly, even the increased circulation time and improved tissue penetration of PEG-Gem did not significantly increase expected adverse side effects as compared to standard Gem treatment (**Fig. 6A & B**).

## Discussion

Here we validate the concept of a multimodal therapy for comprehensively targeting the diverse cell compartments in pancreatic cancer using a representative set of almost 200 subcutaneous and orthotopic whole-tissue primary tumor xenografts, making this one of the largest investigations in the cancer stem cell field. Tumors were selected based on their previously described diverse response to gemcitabine treatment [7]. Chemotherapy and radiation primarily target differentiated cancer cells, and while these therapies induce apoptosis and cell death in tumor cells, a population of cancer stem cells is highly resistant [8], [9], [18], [24], survives the standard therapy, and maintains the ability to re-populate a tumor in all its heterogeneity. Double treatment combining Gem and the new Smoothened inhibitor SIBI consistently prolonged survival in mice transplanted with tumors. Importantly, however, only in mice treated with triple therapy cancer stem cells were virtually completely abrogated, and we observed a long-term disease stabilization or regression, and subsequent long-term survival. In this combination regimen, the treatment effect of conventional Gem could be further enhanced by the use of PEGylated Gem via enhancing its bioavailability.

At the histological level, pancreatic cancer is characterized by very dense stroma and poor vascularization. Olive et al. showed in a genetically engineered mouse model of pancreatic cancer that the stroma is strongly dependent on hedgehog signaling, and inhibition of the hedgehog pathway with smoothened inhibitors leads to “preferential” killing of stromal cells and increased vessel density [21], thus making tumor cells more accessible to therapeutic intervention. While these observations were obtained in a mouse model of pancreatic cancer, we have more recently shown that also in patient-derived whole-tissue xenografts co-treatment with a smoothened inhibitor significantly increases drug delivery [17] and markedly reduces tumor-associated stroma formation. Importantly, we now appreciate that the stroma not only hampers drug delivery [21], but also provides a supportive niche for cancer stem cells promoting their self-renewal capacity and invasiveness [25]. Thus, elimination or abrogation of the stroma does significantly improve treatment regimens by distinct mechanism, but is only capable of eliminating cancer stem cells if combined with Gem.

However, despite the rather modest response of cancer stem cells to hedgehog pathway inhibition as a single agent, we were able to demonstrate that smoothened inhibitors are still essential in order to successfully eliminate chemoresistant pancreatic cancer stem cells if combined with other stem cell-targeting agents [9], [17]. Specifically, we have previously shown for pancreatic cancer that a combination of chemotherapy and inhibitors of both mTOR and hedgehog signaling eliminates differentiated cells as well as cancer stem cells *in vitro* [9], and that this translates into long-term survival *in vivo*. Recently, Wang et al. provided an important mechanistic link for the combined inhibition of the hedgehog and mTOR pathway. Specifically, the authors demonstrate that mTOR/S6K1 signaling results in phosphorylation of Gli and subsequent expression of downstream targets. Inhibition of both pathways greatly enhanced the pro-apoptosis effect of inhibition of either inhibition alone [26]. In the present study, we now saw a virtually complete elimination of cancer stem cells for this combination therapy in a large and representative set of primary xenografts. Whereas flow cytometry using the surface markers CD133, EpCAM, and CD44 already suggested that the cancer stem cell content was strongly reduced, functional assays (e.g. sphere formation assay) validated that the cells isolated from the explanted tumors indeed were unable to form tumor spheres *in vitro,* strongly suggesting that the cancer stem cell population as the root of the disease, had been effectively targeted by the triple combination.

Even though the results using Gem-SIBI-Rapa were highly consistent and encouraging across a panel of patient-derived tumors, we did observe tumor re-growth in some mice (e.g. PDAC-185 xenografts) and, subsequently, a decrease in the survival of these xenograft-bearing animals. As this might be related to the limited bioavailability of the chemotherapeutic agent as an essential part of this combination therapy, we further advanced our treatment strategy by modifying the chemotherapy. Specifically, Vandana et al. have recently shown that modifying gemcitabine via PEGylation leads to enhanced bioavailability in the circulation as compared to native gemcitabine. Although they have also shown better *in vitro* response of established pancreatic cancer cells using PEGylated gemcitabine as compared to the regular formulation [22], we observed no significant differences between Gem and PEG-Gem *in vitro* at the level of cancer stem cell content or induction of apoptosis or cell death using xenograft-derived primary cells. While these data are not surprising as drug delivery and availability is not a critical issue *in vitro*, the results clearly emphasize the importance of utilizing primary cancer tissue for further *in vivo* evaluation of drug efficacy.

Indeed, we were then able to validate and extend this concept to the *in vivo* setting by showing that PEG-Gem treatment significantly delayed the time of tumor progression by 72 days. This enhanced treatment response is certainly impressive as it represents more than half of the study period. As expected, however, tumors ultimately progressed resulting in virtually no difference in median survival of the PEG-Gem treated mice compared to mice treated with traditional Gem alone. While these data confirm that PEGylation of Gem does indeed improve drug availability and delivery, respectively, by enhancing the circulation time and tissue penetration, as expected, PEG-Gem alone is clearly not sufficient to overcome the chemoresistance of cancer stem cells. Therefore, we next investigated the effects of replacing regular Gem with PEG-Gem in our multimodal approach for targeting pancreatic tumors, which had originally responded to Gem+SIBI+Rapa treatment, but eventually relapsed under this specific treatment regimen. Intriguingly, using the PEG-Gem+SIBI+Rapa combination we not only observed virtually complete tumor regression, but most importantly we obtained 100% survival throughout the 125d study period in this highly therapy-resistant tumor. While this observation does not exclude later re-growth as seen in PDAC-Pax22, these data are very promising and are consistent with the notion that further improving the formulation of the combined drugs is mandatory for extending *in vitro* findings to the much more complex *in vivo* setting.

The utilized new smoothened inhibitor SIBI-C1 (Siena Biotech, Italy) was also highly effective *in vivo*, as can be seen by reduced tumor growth in combination with Gem and thus significantly enhanced survival time. Furthermore, SIBI can be safely administered *in vivo*, as we saw no adverse effects on total body weight or white blood cell counts. Importantly, this is well in line with previous observations using other smoothened inhibitors [9], [15]. The Gem+SIBI+Rapa combination therapy also showed no significant toxicity compared to Gem treatment alone during the course of the experiments. PEG-Gem+SIBI+Rapa combination treatment, while much more effective *in vivo*, only slightly, but non-significantly decreased white blood cell counts and had no effect on the body weight of the animals as compared to respective controls. It is important to note, however, that the healthy and relatively young mice used for this study are likely more capable of compensating for putative adverse effects on the normal stem cell compartments during triple therapy treatment. Therefore, it will be important to further validate the safety of this treatment regimen in human patients in order to ultimately apply it to the mostly aged and moribund patients suffering from pancreatic cancer.

In conclusion, here we provide compelling evidence for the efficacy of a multimodal therapy targeting differentiated cells as well as cancer stem cells in pancreatic cancer, resulting in long-term survival in mice. Thus, these data confirm and expand previous findings from our laboratory in a very large cohort of animals with patient-derived pancreatic cancer xenografts [9], [15], [17]. In addition, we also offer a new and novel perspective on how to further improve current therapeutic approaches by modifying the molecular structure of the mandatory chemotherapeutic agents using PEGylation. Taken together, these findings should significantly impact the future development of new anti-pancreatic cancer therapeutics and/or treatment modalities.

## Materials and Methods

### Tumor Samples

After patients' informed consent had been obtained, excess tissues from resected pancreatic carcinomas was xenografted at Johns Hopkins Medical Institutions (JHMIRB: 05-04-14-02 “A Feasibility Study for Individualized Treatment of Patients with Advanced Pancreatic Cancer”) and Hospital de Madrid - Centro Integral Oncológico Clara Campal (FHM.06.10 "Establishment of bank for tumors and healthy tissue in patients with cancer”), respectively, under the indicated Institutional Review Board-approved protocols [7]. Briefly, excess tumor tissues not needed for clinical diagnosis during routine Whipple resections performed by surgeons that were not involved in the present study were subsequently implanted into immunocompromised mice. All patient information was made anonymous by removal of any information, which identifies, or could lead to the identification of the patient. None of the patients had undergone neoadjuvant radiation or chemotherapy prior to resection of the tumor.

### Animal Experiments

All animal experiments were conducted in accordance with institutional guidelines and were approved by the Institutional Animal Care and Use Committee of the CNIO (Protocol PA34/2012– “Xenotransplant model for human pancreatic cancer”). Animals were housed and maintained in laminar flow cabinets under specific pathogen-free conditions. Briefly, 8 mm^3^ pieces of primary, *in vivo* expanded pancreatic cancer tissue pieces were either orthotopically or subcutaneously implanted into the pancreas of 6–8 weeks old female nude mice (Harlan Europe) as described previously [7], [9], [27]. For each treatment group, ≥10 tumors were implanted. Tumor size and body weights of all animals were measured weekly. Size of the subcutaneous tumors was measured by caliper and calculated as length×width×depth. Orthotopic tumors were measured with a dedicated small-animal ultrasound system (Vevo770, Visualsonics, Toronto, Canada), and size was calculated as (length×width^2^)/2. Survival was defined as the time point when tumors reached 1 cm^3^ and mice had to be removed from the study. White blood cell counts were performed with an Abacus Junior Vet hematology analyzer (Diatron, Lenexa, Kansas).

### Allocated Treatments

Gemcitabine was purchased from Lilly (Indianapolis, Indianapolis), dissolved in sterile water and administered twice a week (125 mg/kg i.p.) for 60 days. Rapa (5 mg/kg; Wyeth, Philadelphia, Pennsylvania) was orally administered via the drinking water as described previously [28]. SIBI and Rapa were administered for 21 days. SIBI-C1 (SEN826) was kindly provided by Siena Biotech S.p.A. (Siena, Italy). The characteristics of the compound are similar to that of SEN794 and SEN450, some chemical properties were ameliorated in each of the compounds. SEN450 has previously been used and characterized extensively in *in vitro* and *in vivo* tumor models of glioblastoma [20]. SIBI-C1 was dissolved in a 1∶1 mixture of NaCl and polyethyleneglycol (Sigma Aldrich, St. Louis, Minnesota), and administered at 300 mg/kg by daily oral gavage. At this dose, SIBI-C1 was found to strongly inhibit the expression of the Hedgehog target genes GLI-1 and PTCH, comparable to the inhibitory effects of GDC-0449, in a subcutaneous medulloblastoma model derived from Patch+/− mice (unpublished data Siena Biotech). For *in vitro* experiments, SIBI-C1 was dissolved in DMSO and used at a concentration of 10 µM.

PEGylated gemcitabine was synthesized by Sahoo and colleagues as described previously and with modifications [22], and was administered analogous to regular gemcitabine. The PEGylated gemcitabine was synthesized by conjugating gemcitabine to HOOC-PEG-COOH in dimethylsulfoxide (DMSO), in the presence of triethylamine (TEA). Briefly, HOOC-PEG-COOH (0.1 mM) was dissolved in 2.5 ml of DMSO to which TEA (0.05 ml) was added. Further, NHS (100 mM) and EDC (400 mM) were added to the above solution and the reaction mixture was stirred for 30 min. Later, the synthesized PEG-(NHS)2 was coupled to gemcitabine. In brief, the gemcitabine (0.4 mM) was dissolved in 500 µl and added drop wise to the PEG-(NHS)_2_ solution in the presence of 2 mM TEA (PEG-NHS/Gemcitabine/TEA molar ratio = 1∶4∶20). The reaction mixture was then kept on constant magnetic stirring overnight at room temperature. The reaction mixture was later subjected to dialysis using a dialysis membrane (MWCO: molecular weight cut-off = 3.5 kDa) against distilled water to remove free and unreacted gemcitabine. Subsequently, the dialyzed solution was freeze-dried using a lyophilizer (Labconco, Kansas City, Montana) at a temperature of −48°C and 0.05 mbar to obtain the powdered form of the conjugate. The characterization of the PEGylated gemcitabine was performed as described previously [22].

### RNA Preparation and RT-PCR

Total RNAs from human primary pancreatic cancer cells and spheres were extracted with TRIzol kit (Life Technologies Inc.) according to the manufacturer’s instructions. One microgram of total RNA was used for cDNA synthesis with SuperScript II reverse transcriptase (Life Technologies Inc.) and random hexamers. Quantitative real-time PCR was performed using SYBR Green PCR master mix (Qiagen), according to the manufacturer’s instructions. The list of utilized primers is depicted in **Table S1 in File S2**.

### Western Blot Analysis

Total protein extracts were obtained using M-PER Mammalian Protein Extraction Reagent (Thermo Scientific) supplemented with phosphatase and protease inhibitors. Pellets were incubated during 1 h in lysis buffer at 4°C, and centrifuged at 13,000 rpm during 10 min at 4°C. Total protein concentration was measured with BCA Protein Assay kit (Pierce) and 25–100 mg protein were separated by SDS/PAGE and transferred to PVDF membranes. Upon antibody incubation, membranes were visualized by enhanced chemoluminescence (Amersham). GAPDH was used as a loading control. A complete list of used antibodies is included in **Table S2 in File S2**.

### Flow Cytometry

To characterize pancreatic cancer stem cells, the following antibodies were used: anti-CD133/1-APC (clone AC133 Miltenyi Biotech, Bergisch Gladbach, Germany), anti-CD44-PE anti-EpCAM FITC (both Becton Dickinson, Heidelberg, Germany) or appropriate isotype-matched control antibodies. Samples were analyzed by flow cytometry, using a FACSCanto II (BD), and data were analyzed with FloJo 9.4.4 (Treestar, Ashland, Oregon). Apoptosis and cell death analyses were performed using DAPI and an Annexin V fluorescein isothiocyanate (FITC) staining kit (BD).

### Histology

Formalin-fixed, paraffin-embedded tumor sections were stained with a CK19 antibody (1∶500, Dako, Carpinteria, CA), and then visualized with a rabbit anti-mouse and anti-rabbit HRP-conjugated antibody (both Epitomics). Nuclear counterstaining was performed using Hematoxylin. The stroma quantification was performed by two independent investigators, one an experienced pathologist (E.G.).

### Cell Culture

For *in vitro* studies, tumors were enzymatically digested with collagenase and pancreatic cancer adherent cell and sphere cultures were generated and expanded as previously described [17], [18]. Five thousand cells per milliliter were seeded in ultra-low attachment plates (Corning B.V., Schiphol-Rijk, Netherlands) and monitored for sphere formation capacity over the course of 14 days. Spheres were defined as 3-dimensional multicellular structures of approximately 40 µm or larger. For in vitro treatment, 10^5^ cells per well were seeded in 6-well plates, treated with either standard gemcitabine or PEG-Gem at a concentration of 100 ng/mL after 24 h, and analyzed on day 3 by flow cytometry to detect apoptosis and cancer stem cell content.

### Statistical Analysis

Results for continuous variables are expressed as means ± standard error of the mean (SEM) unless stated otherwise. Overall comparison of continuous variables was performed with the Kruskal-Wallis test followed by post hoc pairwise comparison using the Mann-Whitney U test. Survival was compared using a Log Rank test. P values <0.05 were considered statistically significant. All analyses were performed with SPSS 19 (SPSS Inc., Chicago, IL).

## Supporting Information

File S1 **Figure S1A: Effect of SIBI-C1 on Hedgehog pathway gene expression.** Fold increase mRNA expression levels of SHH and GLI2 in gemcitabine resistant primary cancer cells (PDAC-265 left panel, PDAC-354 right panel). **Figure S1B: Effect of combination therapy on tumor composition.** Representative histological pictures showing stroma content in the respective treatment groups in gemcitabine resistant subcutaneously implanted tumors (PDAC-185, upper panel), (354, lower panel).
(TIF)

File S2 **Table S1: Utilized qRT-PCR primers. Table S2: Utilized antibodies.**
(TIF)
