# Supplementary figures and images for: Multimodal Treatment Eliminates Cancer Stem Cells and Leads to Long-Term Survival in Primary Human Pancreatic Cancer Tissue Xenografts
Source: PLoS One. 2013 Jun 18;8(6):e66371. doi: 10.1371/journal.pone.0066371 (PMC3688976; doi:10.1371/journal.pone.0066371)

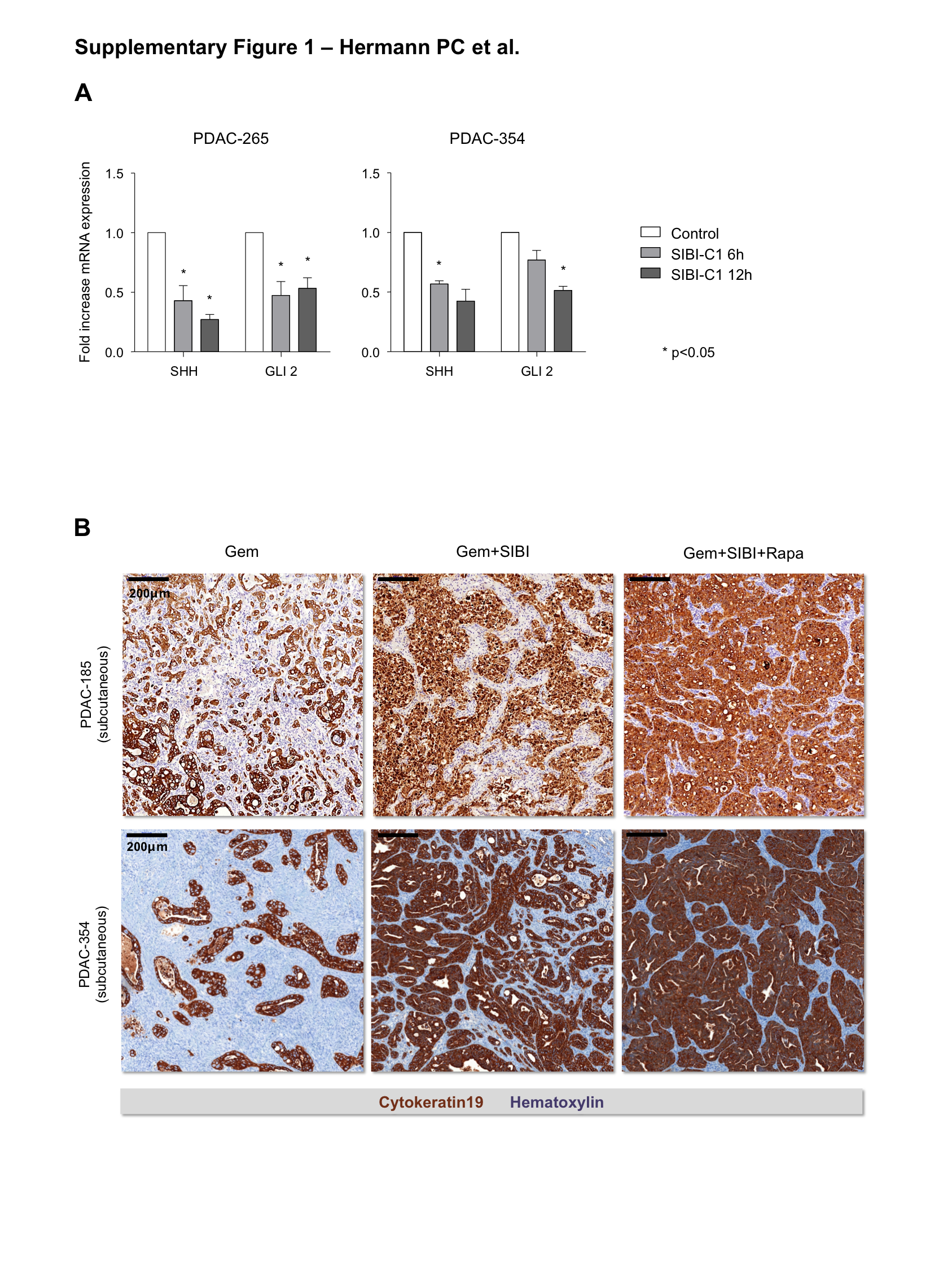

Supplement: File S1 — Figure S1A: Effect of SIBI-C1 on Hedgehog pathway gene expression. Fold increase mRNA expression levels of SHH and GLI2 in gemcitabine resistant primary cancer cells (PDAC-265 left panel, PDAC-354 right panel). Figure S1B: Effect of combination therapy on tumor composition. Representative histological pictures showing stroma content in the respective treatment groups in gemcitabine resistant subcutaneously implanted tumors (PDAC-185, upper panel), (354, lower panel). (TIF) [file pone.0066371.s001.tif]

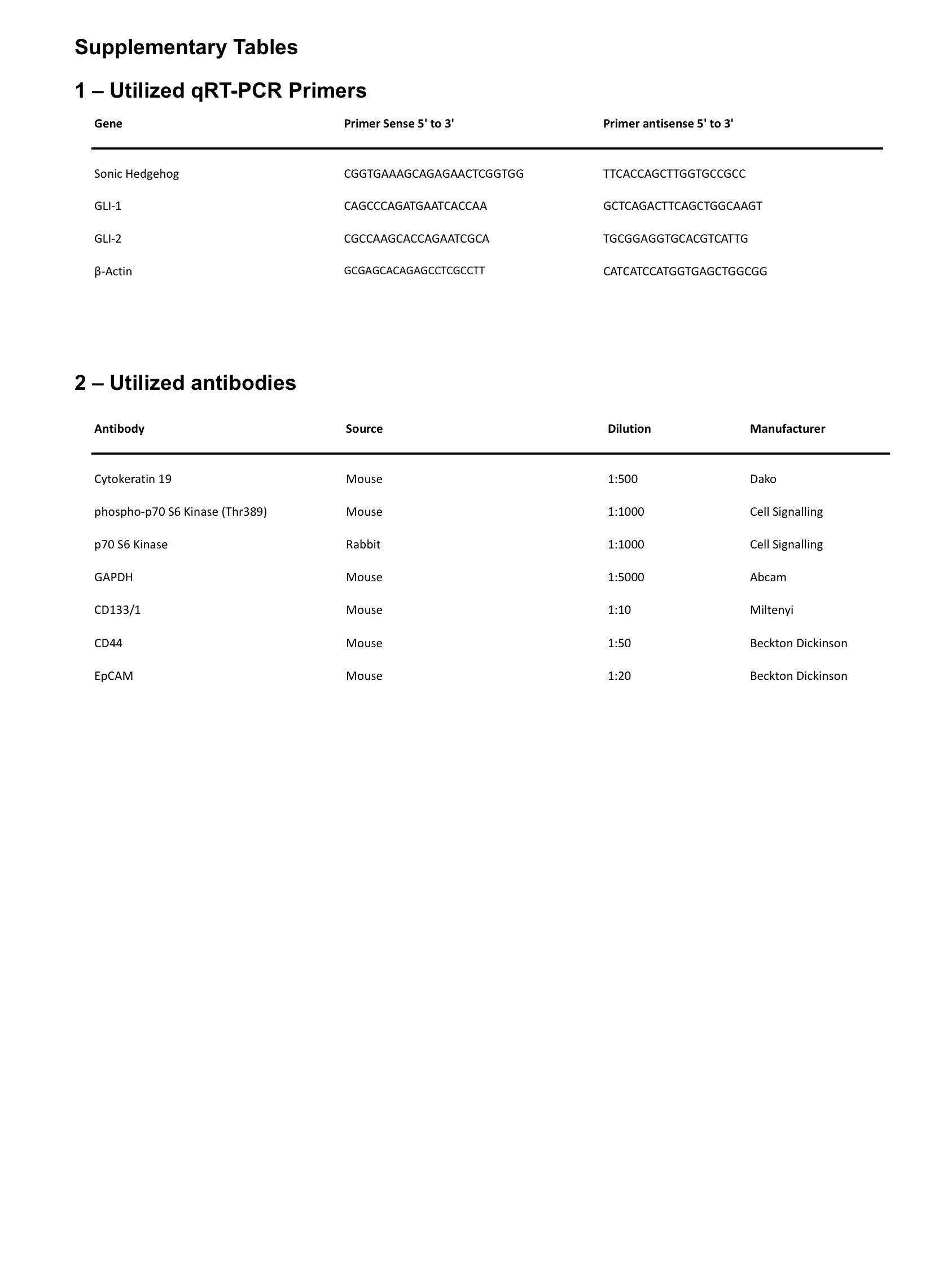

Supplement: File S2 — Table S1: Utilized qRT-PCR primers. Table S2: Utilized antibodies. (TIF) [file pone.0066371.s002.tif]
